# Effect of Moxibustion on Hyperhomocysteinemia and Oxidative Stress Induced by High-Methionine Diet

**DOI:** 10.1155/2020/3184785

**Published:** 2020-03-09

**Authors:** Hao Wang, Lue Ha, Xin Hui, Yao Lin, Rui He, Zhao Baixiao

**Affiliations:** Beijing University of Chinese Medicine, Beijing, China

## Abstract

**Objective:**

The aim of this study was to assess the effects of moxibustion on the animal model of oxidative stress and cardiovascular injury induced by high-methionine diet (2% methionine and 3.5% fat on the basis of ordinary maintenance feed) during 12 weeks.

**Methods:**

53 mice were divided into four groups: mice in the Control group (*n* = 8), mice in the Met group (*n* = 8), mice in the Met group (*n* = 8), mice in the Met group (*n* = 8), mice in the Met group (

**Results:**

Compared with the Met group, our results indicated that through moxibustion intervention, the content of serum Hcy and its intermediate metabolite SAH can be reduced to a certain extent, and SOD, HO-1, and ox-LDL can be increased.

**Conclusion:**

This study showed moxibustion's ability to enhance the body's antioxidation and protect vascular endothelial function, thus playing an early role in the prevention and treatment of atherosclerosis.

## 1. Introduction

In addition to many traditional risk factors (such as hypertension, diabetes, hyperlipidemia, and smoking), more and more studies have focused on the relationship between homocysteine (Hcy) and atherosclerosis (AS) [1, 2]. Hyperhomocysteinemia (HHcy) is an independent risk factor for atherosclerosis [3, 4]. HHcy is defined as blood Hcy >15 mmol/L. HHcy can resist the growth of endothelial cells and endothelial dysfunction or impaired re-endothelialization, affect the normal relaxation function of endothelium, and accelerate the hyperplasia and thickening of the intima and smooth muscle cells, resulting in endothelial dysfunction [5, 6]. About 75% of the nearly 300 million hypertensive patients in China are accompanied by HHcy, and the risk of cardiovascular disease increases by 1.6–1.8 times with the increase of serum Hcy level by 5 *μ*mol/L in a dose-dependent manner [7, 8].

According to the formation mechanism of HHcy, the current expert guidelines recommend folic acid and vitamin B12 supplementation for treatment [8–12], but meta-analysis results show that folic acid supplementation for more than three years is necessary to reduce the risk of stroke [13]. Therefore, there is a necessary need to find a treatment method which can be used for people's daily health care and exerts definite therapeutic effects. According to the idea of “preventive treatment of diseases” in traditional Chinese medicine, it is possible to choose the traditional nonpharmacological interventions—moxibustion.

Moxibustion therapy has lasted for thousands of years. Moxa velvet or other herbs is ignited, which directly or indirectly stimulates acupoints or specific parts of the body surface through burning, fumigation, and ironing, and with the help of the mild heat produced by burning moxibustion materials and the medicinal effect of moxa smoke occurs transmission of meridian acupoints, warming and ventilating blood circulation, activating collaterals, and removing arthralgia, so as to achieve disease prevention, treatment, rehabilitation, and health care [14–17]. It is a traditional method of external treatment for the purpose of health care.

Previous clinical trials of our team have proved that moxibustion can reduce the content of serum Hcy in patients with hypertension, and through a series of animal experiments on atherosclerosis, it is concluded that moxibustion may treat cardiovascular diseases by affecting blood lipid metabolism [18], platelet coagulation system [19], inflammatory response of the body [20], protection and maintenance of vascular endothelial cell function, and other ways. Moxibustion has therapeutic effect on atherosclerotic lesions and alleviates their pathological process, alleviates the development of atherosclerotic plaques, plays a benign regulatory role in lipid metabolism, reduces blood lipid, promotes cholesterol outflow from lesions, improves liver lipid metabolism function, inhibits platelet activation, alleviates the pathological changes of atherosclerosis, and plays a role in the prevention and treatment of atherosclerosis.

In this study, moxibustion and moxibustion smoke were used as intervention methods, and C57BL/6J mice fed with high-methionine diet were used as modeling methods and observation objects to further explore the possible mechanism of moxibustion and moxibustion smoke in the treatment of HHcy and the preventive effect on early AS.

## 2. Methods

High-methionine feed was purchased from Beijing Keao Xieli Feed Co., Ltd. (feed was added with 2% methionine and 3.5% fat on the basis of ordinary maintenance feed [21]). The applied doses were selected based on the literature data which showed that moderate hyperhomocysteinemia (plasma tHcy levels of 18–60 *μ*mol/L) can be achieved by increasing the total methionine content up to 12 to 20 g/kg in diet.

### 2.1. Animals and Treatment

Experimental preparations and protocols were reviewed and approved by the Institutional Animal Care and Use Committee of the Beijing University of Chinese Medicine (BUCM-4-2017010606-1006).

In our experiment, we used 8-week-old healthy female SPF C57BL/6J mice (*n* = 53; BW = 20–25 g; Beijing Vital River Laboratory Animal Technology Co., Ltd. no. 11400700213655). All mice were fed in the SPF Barrier Laboratory of Beijing University of Chinese Medicine. Animals were acclimatized for 7 days to laboratory conditions. Animals were kept in conventional plastic cages (four or five mice per cage) in a light and temperature-controlled room with a 12/12 h light-dark cycle, and ambient temperature and relative humidity were maintained at (22 ± 2)°C and 50%–60%, respectively. Animals had free access to water and food through the experimental period.

53 mice were divided into four groups: mice in the Control group (*n* = 8), mice in the Met group (*n* = 15), mice in the Met + Moxibustion group (*n* = 15), and mice in the Met + Smoke group (*n* = 15).

Met + Moxibustion (MM) group: the mice were fed with a high-methionine diet. These mice were grabbed and fixed with a fixator to fix the head, tail, and limbs. The mice were put into the fixator and erected. The bottom of the fixator was equipped with holes, which exposed the midpoint of the chest, and the moxa sticks were set under the chest of the mice, so that the heat was transmitted to the midpoint of the chest of the mice through the holes at the bottom of the fixator. In the process of moxibustion, the height of the moxibustion stick is constantly adjusted to keep the temperature of moxibustion basically constant. Moxibustion intervention lasted for 20 minutes, once a day, 6 times a week, and for 12 weeks. Mice were executed at the end of 12 weeks.

Met + Smoke (MS) group: mice were fed with a high-methionine diet and captured and fixed like the moxibustion group. Then, the fixator was placed in a self-made glass jar, and the moxa stick was ignited. The concentration of moxa smoke in the glass jar was measured by a microcomputer laser dust monitor. The concentration of moxa smoke was kept at 10–15 mg/m^3^ (20 min/day; 6 times/week).

  Control group: normal diet feeding and no intervention.
  Met group: mice were fed with a high-methionine diet and captured, and the fixation method was the same as the moxibustion group.

After 12 weeks, mice were euthanized with 1%, 50 mg/kg pentobarbital sodium under the guidance of Euthanasia Guideline (AVMA). Also aorta and blood samples were taken for histological and biochemical analysis. Blood samples were collected from the orbital venous plexus about 1.5–2 ml and placed in a centrifugal tube for 1 hour. After centrifugation, blood samples were centrifuged for 15 minutes. After centrifugation, the upper serum was stored in a refrigerator at −20°C for testing.

### 2.2. Biochemical Analyses in Serum and Aorta Tissue

Hcy and SAH in serum, SOD, ox-LDL, eNOS, and HO-1 in the aortic homogenate were determined by the enzyme-linked immunosorbent assay (Elisa).

The aorta tissue samples were washed and fixed in 4% paraformaldehyde for 24 hours and then stained with hematoxylin-eosin. Then, the samples were embedded in paraffin and sectioned at 5 *μ*m thickness. The sections were stained by the hematoxylin-eosin method. Optical microscope Motic BA400 and Motic Images Advanced 3.2 imaging software systems were used for tissue slice analysis, and 10x and 40x of the photos were selected.

### 2.3. Statistical Analysis

The experimental results were analyzed by SPSS 20.0 statistical software. All the data were tested by the normality test and homogeneity test of variance. The measured data which accorded with normal distribution and homogeneity of variance were expressed by mean ± standard error (SE) and analyzed by one-way ANOVA. When there were significant differences between groups, multiple comparisons between groups were made by the LSD method. *P* < 0.05 was considered to have statistical significance. For the data of non-normal distribution, the median (quartile distance) *M* (IQ) was used and the nonparametric test (Mann–Whitney *U*) was used to compare the data between groups; *P* < 0.05 was the significant difference.

## 3. Results

### 3.1. Hcy and SAH in Serum

**Figure 1:**
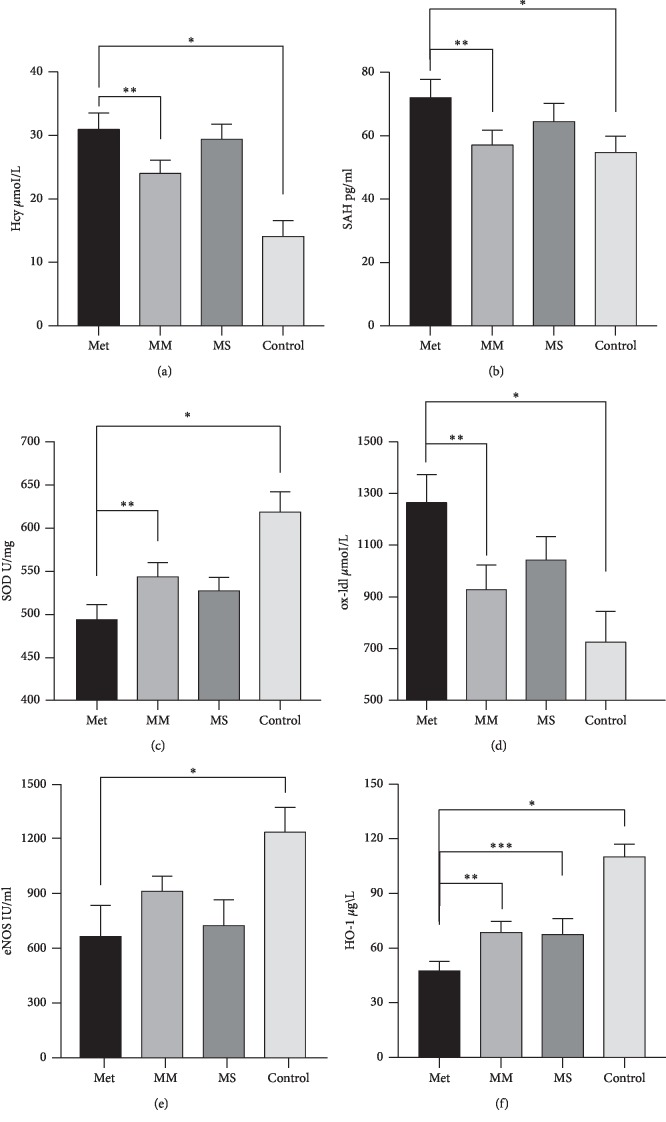
Hcy and SAH in serum, SOD, ox-LDL, eNOS, and HO-1 in the aortic homogenate.

The content of Hcy in serum of mice in each group was not identical, showing the trend of Met group > MS group > MM group > Control group. Results in Figure 1(a) show that compared with the Control group, the serum Hcy content of the three groups (Met group, MM group, and MS group) fed with high-methionine diet increased significantly (*P* < 0.01), and the mean content of the Met group and the MM group was twice as high as that of the Control group; compared with the Met group, the serum Hcy content of the MM group was significantly lower (*P* < 0.05).

The serum S-adenosine homocysteine (SAH) content in each group was different. Kruskal–Wallis *H* test showed that *P*=0.049, and the mean ranking was Met group > MS group > MM group > Control group. The results are shown in Figure 1(b). The results showed that compared with the Met group, the MM group and the Control group had a significant difference (*P*=0.022 and *P*=0.041), and there was no significant difference between the MS group and the Met group.

### 3.2. Antioxidant Status in Aorta Tissue

The superoxide dismutase (SOD) in the aortic homogenate of each group was significantly different, showing the trend of the Control group > MM group > MS group > Met group. Results are shown in Figure 1(c), and compared with the Control group, the SOD content in the aorta of the Met group, MM group, and MS group decreased significantly; compared with the Met group, the SOD content in the MM group was *P*=0.021, the difference was significant.

There was a significant difference in oxygenized low-density lipoprotein (ox-LDL) in the aortic tissue homogenate of each group, showing the trend of Met group > MS group > MM group > Control group. The results are shown in Figure 1(d). Compared with the Control group, the content of ox-LDL in the MS group and the Met group increased significantly; compared with the Met group, the content of ox-LDL in the MM group decreased significantly.

Endothelial nitric oxide synthase (eNOS) in the aortic homogenate of each group was not identical, and the mean value of eNOS in the aortic homogenate showed the trend of Control group > MM group > MS group > Met group. The results are shown in Figure 1(e). Compared with the Control group, the eNOS content of the three groups (Met group, MM group, and MS group) fed with high-methionine diet decreased significantly. But compared with the Met group, there was no significant difference in the MM group and the MS group.

Heme oxygenase-1 (HO-1) in the aortic homogenate of each group was different, showing the trend of Control group > MM group > MS group > Met group. The results are shown in Figure 1(f). Compared with the Control group, the content of HO-1 in the MM group, the MS group, and the Met group was significantly lower; compared with the Met group, the MM group and the MS group were significantly higher, *P* < 0.05.

### 3.3. Histological Analysis

#### 3.3.1. Control Group

**Figure 2:**
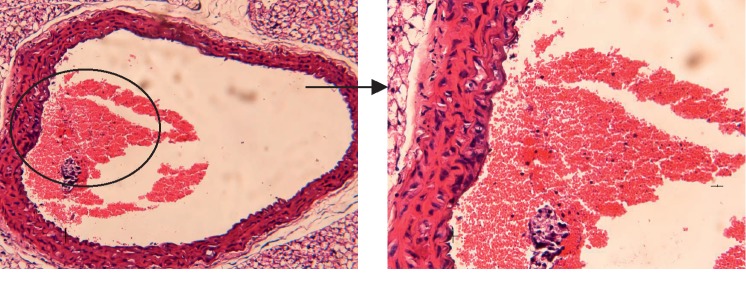
Histological analysis of the aorta tissue in the Control group.

Histological analysis of the aorta tissue in the Control group (Figure 2) showed that the thickness of the vessel wall was normal, and the endothelial cells were intact and the intima was smooth.

#### 3.3.2. Met Group

**Figure 3:**
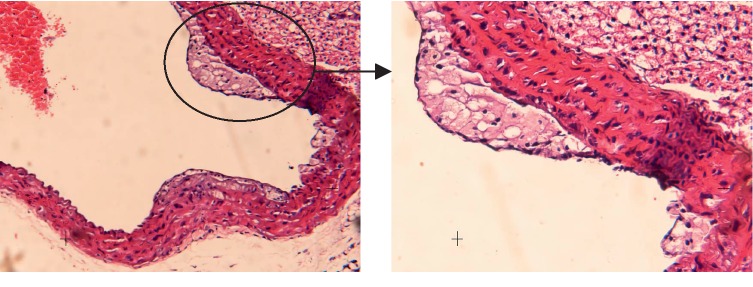
Histological analysis of the aorta tissue in the Met group.

Histological analysis of the aorta tissue in the Met group (Figure 3) showed that the vascular wall was thickened and the fibrous cap on the surface had a large number of smooth muscles and extracellular matrix. The proliferated smooth muscle, macrophages, extracellular lipids, and matrix were under the fibrous cap.

#### 3.3.3. Met + Moxibustion Group

**Figure 4:**
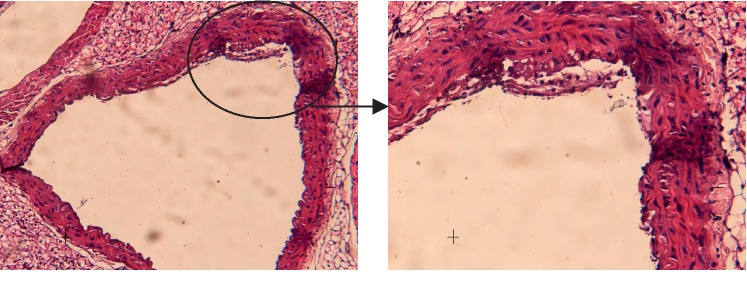
Histological analysis of the aorta tissue in the Met + Moxibustion group.

Histological analysis of the aorta tissue in the Met + Moxibustion group (Figure 4) showed that the thickness of the vascular wall is normal, the endothelial cells are intact, the intima is smooth, and there are a few foam cells gathered under the endothelial cells.

#### 3.3.4. Met + Smoke Group

**Figure 5:**
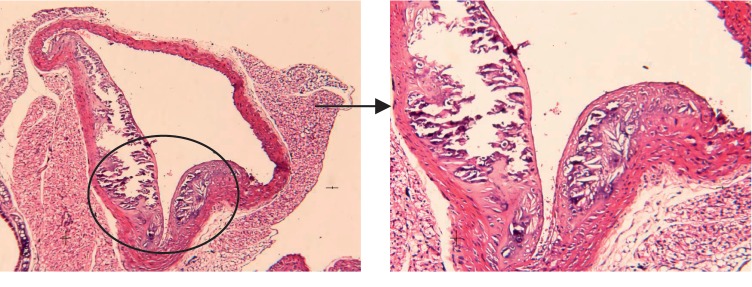
Histological analysis of the aorta tissue in the Met + Smoke group.

Histological analysis of the aorta tissue in the Met + Smoke group (Figure 5) showed the atherosclerosis secondary lesions, the surface of which had a fibrous cap under which is a large number of amorphous necrosis rich in lipids, cholesterol crystallization, and calcification.

## 4. Discussion

Hcy is one of the independent risk factors for atherosclerosis [22, 23]. The pathological increase of Hcy is mainly due to the disorder of metabolic circulation. Excessive methionine intake or dysfunction of excretion and the lack of folic acid (a necessary enzyme in methionine metabolism) are the main factors [1].

Hcy is an intermediate product of the methionine metabolism pathway in vivo. Methionine condenses with ATP to form S-adenosylmethionine (SAM), which is the main methyl donor of the polymethylation reaction in vivo. S-adenosylhomocysteine (SAH) is produced by transmethylation of SAM and then hydrolyzed to form homocysteine (Hcy) and adenosine [24, 25]. SAM and SAH can activate cystathionine beta-synthase (CBS) and inhibit methyltetrahydrofolate reductase activity, resulting in further increase of Hcy production [26–28]. In a German, Austrian, and Swiss consensus document [29], according to various computational models, the reduction of hyperhomocysteinemia can theoretically prevent up to 25% of cardiovascular events [6, 30, 31]. It is suggested that the target plasma homocysteine level should be less than 10 mmol/L.

HHcy can induce oxidative stress, initiate endothelial and lipid peroxidation, damage endothelial cells (EC) and mitochondrial DNA, further cause EC dysfunction, and promote EC apoptosis and plaque formation by inhibiting the activity of antioxidant enzymes and releasing reactive oxygen species (ROS) by its own oxidation. In our previous clinical study, we found that moxibustion has a good effect on reducing serum Hcy in patients with hypertension.

Vitamins such as folic acid and vitamin B12 are cofactors of transmethylase in the homocysteine metabolism pathway. Folic acid is one of the few methods to reduce Hcy, and it has been recommended to be supplemented in combination with Chinese hypertension guidelines. However, epidemiological data [32–34] show that vitamin supplementation cannot reduce the incidence of cardiovascular events. Meta-analysis [35, 36] shows that vitamin supplementation cannot reduce the incidence of cardiovascular events. Folic acid supplementation for more than three years is needed to reduce the risk of stroke. A randomized double-blind study published in JAMA [37] involved 5442 women with cardiovascular risk factors in the United States. After 7.3 years of treatment and follow-up, the results showed that folic acid and vitamin B combination tablets had no therapeutic effect on total cardiovascular events in high-risk women. Therefore, it is possible to find an intervention method which can be used in daily health care and has a wide range of therapeutic effects. According to the idea of “pretreatment of disease” in traditional Chinese medicine, it is possible to choose moxibustion, a traditional nondrug therapy.

In this study, we show experimental evidence that moxibustion can reduce hyperhomocysteinemia induced by hypermethionine diet, which is consistent with previous clinical studies. In addition, it can protect the cardiovascular system by enhancing the antioxidant capacity of mice, which is consistent with the previous moxibustion to reduce the damage caused by oxidative stress.

In order to establish the animal model of hyperhomocysteinemia, C56BL/6 mice (8 weeks old) were fed with high-methionine diet for 12 weeks. We found that a large number of foam cells accumulated in the endothelial cells of the aortic wall, showing cholesterol crystallization and inflammatory infiltration. The hepatocytes were swollen and bulky, the nuclei were obvious, and the boundary between the cells was unclear. Compared with the Met group, SOD and HO-1 in the Met + Moxibustion group increased significantly and eNOS did not show significant difference, but there was a certain upward trend; ox-LDL decreased significantly. It can be seen that moxibustion can restrain the oxidative stress effect caused by Hcy and reduce the oxidative products produced by ROS reaction, which is consistent with previous studies.

The results showed that serum Hcy increased in mice fed with high-methionine diet, which was significantly different from those fed with the normal diet in the Control group. The model of hyperhomocysteinemia was successfully established. Compared with the model group, the levels of serum Hcy and SAH in the moxibustion group have decreased, which has achieved a certain therapeutic effect and reduced the risk of disease, but it has not been reduced to the normal level. In the follow-up study, the combination of moxibustion and folic acid treatment group can be set up to explore the prevention and treatment effect of moxibustion combined with medicine.

The main mechanism of hyperhomocysteinemia on the cardiovascular system is to promote the production of oxygen-free radicals, damage vascular endothelial cells, and cytotoxicity. Studies have shown that [10, 38, 39] Hcy promotes the production of reactive oxygen species (ROS) in the human aortic smooth muscle cells (HASMC) in a concentration-dependent manner. Hcy has a high active thiol group which is easy to self-oxidize through the disulfide bond and other free radicals. In addition, Hcy can increase the expression of NADPH oxidase and produce a large amount of ROS to promote oxidative stress. Higher concentration of Hcy can increase the level of oxidative damage of human umbilical vein endothelial cells (HUVEC), and the addition of catalase can reduce the toxicity of Hcy [40, 41].

In addition to inducing ROS and other oxides, Hcy also affects the antioxidant capacity of EC [42–44], significantly lowering the expression of antioxidants SOD and GSH-Px, resulting in the increase of H_2_O_2_ and serious damage to the body's antioxidant capacity. Some studies have shown that [45] the content of Hcy is positively correlated with the content of MDA in response to oxidative stress, and the NO level is significantly lower than that in the normal population, which briefly reflects the endothelial dysfunction.

According to the theory of traditional Chinese medicine, atherosclerosis is caused by the accumulation of human metabolic waste in the blood vessels, which forms blood stasis and phlegm turbidity, so it should be treated by adjusting the balance of Yin and Yang, promoting blood circulation, and removing blood stasis. In this study, the selected treatment site in moxibustion therapy is the classic acupoint Danzhong (CV17) in the chest, the middle point of the sternum, where moxibustion has the function of strengthening the heart function, making the breath smooth, and making the blood vessels healthy and the blood flow smooth. In animal experiments, moxibustion in this position of mice can be observed that moxibustion has therapeutic effect on atherosclerotic lesions, alleviates its pathological process, alleviates the development of atherosclerotic plaques, plays a benign regulatory role in the metabolism of blood lipids, reduces blood lipids, regulates hyperlipidemia and as model animal serum TC, TG, LDL-C, HDL-C, apo-a/apo-B, and atherosclerosis index (AI), upregulates LXR*α*- and ABCA1-mediated cholesterol efflux in the lesion area, and improves the function of liver lipid metabolism [18–20, 46].

From the point of view of hyperhomocysteinemia, this study complements the therapeutic effect of moxibustion, which can further study the effect of moxibustion on reducing the methylation caused by homocysteine.

There are several limitations to the present study. First, the treatment period is set as 12 weeks, but there was no observation of moxibustion in the early period and long-term observation experiment, so we plan to extend the follow-up evaluation time in the future research, so as to understand the treatment effect of moxibustion more comprehensively. In addition, no other detection methods such as immunohistochemistry were used to further observe the metabolic pathway of moxibustion affecting homocysteine because this study first needs to prove the effect consistent with the clinical trial, so it is expected to conduct further mechanism research in the future.

In conclusion, our study provides experimental evidence that moxibustion treatment at CV17 suppresses the progression of atherosclerosis in mice with hyperhomocysteinemia induced by hypermethionine diet. The therapeutic atherosclerotic effect of moxibustion can be achieved by (1) regulating homocysteine metabolism, reducing its pathological increase; (2) improving the body's antioxidant capacity, reducing oxidative stress damage.

## Data Availability

The data used to support the findings of this study are available from the corresponding author upon request.
